# Rosiglitazone blocks first trimester *in-vitro* placental injury caused by NF-κB-mediated inflammation

**DOI:** 10.1038/s41598-018-38336-2

**Published:** 2019-02-14

**Authors:** Leena Kadam, Brian Kilburn, Dora Baczyk, Hamid Reza Kohan-Ghadr, John Kingdom, Sascha Drewlo

**Affiliations:** 1 Department of Obstetrics & Gynecology, Wayne State University School of Medicine, Detroit, MI USA; 2 The Research Centre for Women’s and Infant’s Health, Lunenfeld Tanenbaum Research Institute, Mount Sinai Hospital, University of Toronto, Toronto, Canada; 3 Department of Obstetrics, Gynecology and Reproductive Biology, College of Human Medicine, Michigan State University, Grand Rapids, MI USA

## Abstract

Increased inflammation and abnormal placentation are common features of a wide spectrum of pregnancy-related disorders such as intra uterine growth restriction, preeclampsia and preterm birth. The inflammatory response of the human placenta has been mostly investigated in relation to cytokine release, but the direct molecular consequences on trophoblast differentiation have not been investigated. This study measured the general effects of LPS on both extravillous and villous trophoblast physiology, and the involvement of the transcription factors PPARγ and NF-κB, specifically using 1^st^ trimester explants and HTR-8/ SVneo cell line models. While both proteins are known for their roles in inflammatory pathways, PPARγ has been identified as an important molecule in trophoblast differentiation, suggesting its potential role in mediating a crosstalk between inflammation and trophoblast differentiation. Here, LPS (1 µg/ml) exposure of first trimester placental villous explants resulted in secretion of inflammatory cytokines, induction of apoptosis and reduction in trophoblast cell proliferation. Additionally, LPS significantly reduced expression of the trophoblast differentiation proteins GCM1 and β-hCG, and increased invasion of the extravillous trophoblast. Activation of PPARγ by Rosiglitazone (10 µM) reversed the LPS-mediated effects on inflammatory cytokine release, trophoblast apoptosis and proliferation compared to controls. Lastly, markers of trophoblast differentiation and invasion reverted to control levels upon activation of PPARγ and concomitant inhibition of NF-κB (either by Rosiglitazone or NF-κB specific inhibitor), revealing a new role for NF-κB in trophoblast invasion. This study reveals a novel PPARγ - NF-κB axis that coordinates inflammatory and differentiation pathways in the human placenta. The ability to reverse trophoblast-associated inflammation with Rosiglitazone offers promise that the PPARγ – NF-κB pathway could one day provide a therapeutic target for placental dysfunction associated with both inflammation and abnormal trophoblast differentiation.

## Introduction

Healthy pregnancy is characterized by dynamic inflammatory changes throughout gestation. A proinflammatory environment at the maternal fetal interface is important for implantation and preliminary stages of placentation^1^. However, several pregnancy disorders, including preeclampsia (PE), intrauterine growth restriction (IUGR), and preterm birth (PTB) that are associated with abnormal placental development, often show pathological levels of both local and systemic inflammation^2–4^. Both PTB and PE placentae have increased pro-inflammatory cytokine release compared to gestational age matched controls^5–10^. In current literature, it is unclear if abnormal placental development and inflammation are linked. Understanding this link would provide insights into the etiologies of these syndromes and might suggest new interventions and management strategies for at risk pregnancies.

*In vitro* studies showed that exposure to inflammatory stimuli induces pro-inflammatory cytokine secretion from trophoblast cells^11,12^. Pro-inflammatory cytokines like TNF-α and IL-6 induce trophoblast cell apoptosis and affect invasion. Conflicting results obtained by various groups are largely inconclusive, which can be attributed to the diverse models used in these studies^13–15^. The pro-inflammatory transcription factor, nuclear factor of kappa light polypeptide gene enhancer in B-Cells (NF-κB), was implicated in regulating placental growth factor (PlGF), a protein known for its role in placental angiogenesis and trophoblast proliferation, suggesting a potential role of inflammatory mediators in trophoblast function^16,17^. However, the molecular link between inflammation and trophoblast differentiation is currently unknown.

This gap in knowledge is further obfuscated by the complexity of trophoblast differentiation process in itself, which is a tightly regulated process that involves numerous crucial proteins and transcription factors^18^. The transcription factor peroxisome proliferator-activated receptor gamma (PPARγ), that is known for its role in energy metabolism and anti-inflammatory processes, has emerged as a player in trophoblast lineage differentiation and placental function in both mice and human models^19–25^. PPARγ mice knockouts die *in-utero* due to gross placental abnormalities which were rescued by replenishing PPARγ in the trophectoderm lineage, affirming its role in placental development^26–28^. Aberrant PPARγ levels/activity have also been associated with human pathologies such as gestational diabetes (GDM), preterm birth and IUGR^29,30^. Further, activation of PPARγ (by Rosiglitazone) in a mouse model of inflammation induced preterm birth, rescued premature delivery, reduced inflammation (by repressing NF-κB activity in macrophages) and improved both placental and fetal weights, suggesting its overlapping function in inflammatory and placental development pathways^31,32^. *In-vitro* investigation of human term placentae and gestational membranes showed that activation of PPARγ could reduce LPS-induced cytokine expression, supporting its anti-inflammatory action in the human placenta^33^. However, it remains unclear whether the roles of PPARγ in inflammation and trophoblast differentiation are linked.

In the current study, the effects of inflammation on trophoblast differentiation and the potential role of PPARγ were evaluated in tissue and cell-based models. In 1^st^ trimester placental explant culture and cell-based models. The bacterial LPS lipopolysaccharide (LPS) was used to induce inflammation in combination with Rosiglitazone, as a PPARγ activator^34^. Rosiglitazone, a thiazolidinedione group compound, selectively activates PPARγ. Rosiglitazone (via PPARγ) has been reported to have anti-inflammatory activities in several disease models and *in-vitro* systems^34–38^. We hypothesized that activation of PPARγ by Rosiglitazone would lessen inflammation-mediated effects on trophoblast differentiation and pathophysiology.

## Results

### The effects of Rosiglitazone on endotoxin (LPS)-induced inflammatory cytokine secretion in the first trimester placenta

**Figure 1:**
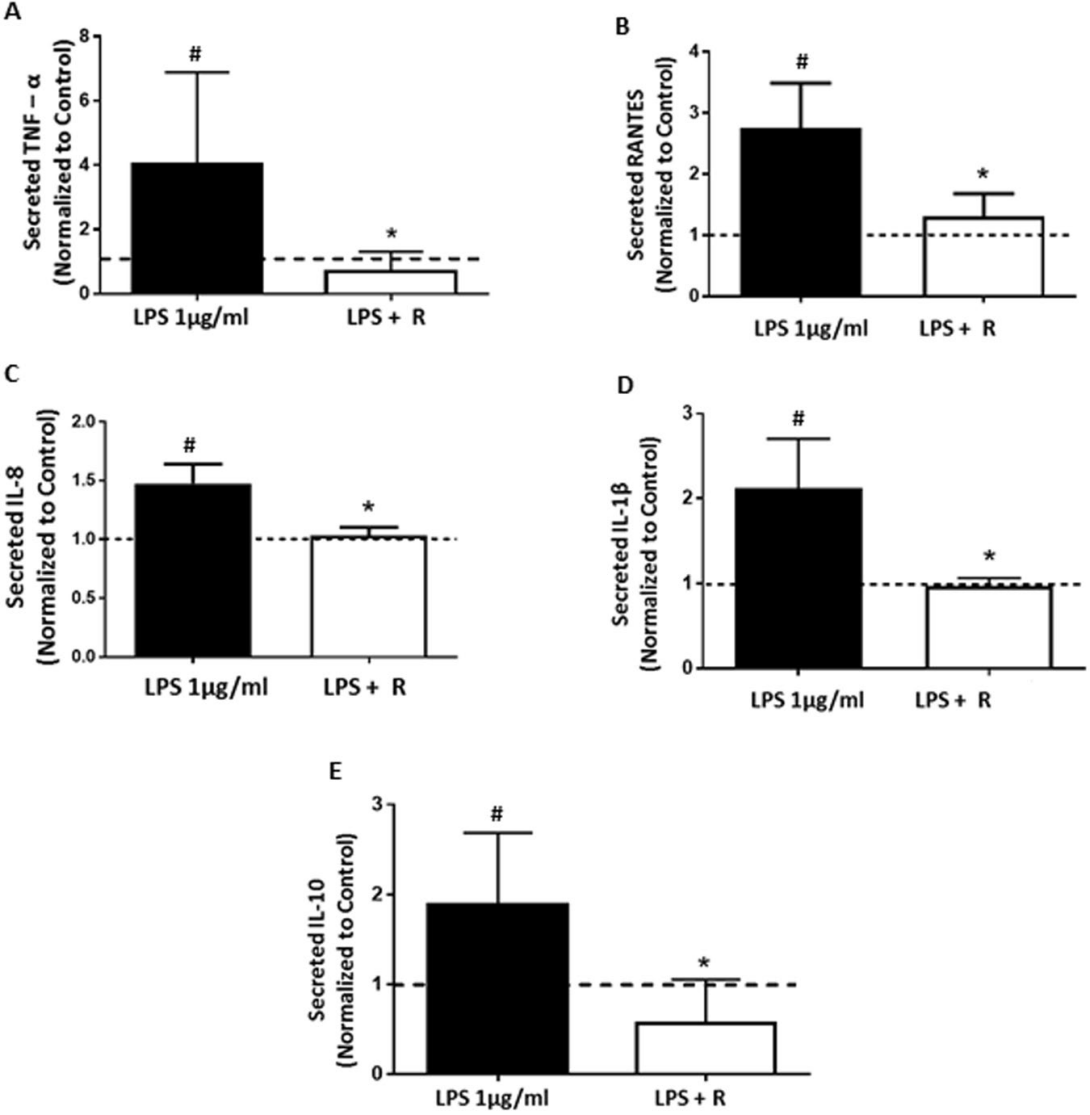
Rosiglitazone treatment reduced inflammatory cytokine secretion in first trimester human placental explant cultures. Cytokine secretion by first trimester human placental explants at 24 hrs of LPS + Rosiglitazone treatment was measured by ELISA. Rosiglitazone treatment significantly downregulated the LPS induced secretion of inflammatory cytokines (**A**) TNF-α, (**B**) RANTES, (**C**) IL-8, (**D**) IL-1 β and (**E**) IL-10. n ≥ 12, For analysis data was normalized to tissue weight and PBS treated control to account for tissue to tissue variations. ‘*’ indicates significance at p < 0.05 when compared to the LPS treated group, ^‘#’^ indicates significance at p < 0.05 when compared to the PBS control group. Dotted line indicates levels in controls.

The inflammatory response of first trimester villous explants exposed to LPS ± Rosiglitazone was assessed using ELISA to quantify inflammatory cytokines in the culture medium. LPS exposure induced inflammatory cytokine secretion from the explants. Compared to controls, media from explants in the LPS group had significantly higher levels of TNF-α (≥3.0-fold, p = 0.034), RANTES (≥2.7-fold p = 0.013), IL-8 (≥1.4fold, p = 0.034) and IL-1 β (≥2.0-fold, p = 0.042) (Fig. 1A–D). Rosiglitazone (Rosi) treatment ameliorated the LPS induced effects. Compared to the LPS group, the LPS + Rosi group had significantly reduced levels of TNF-α (p = 0.009), RANTES (p = 0.0134), IL-8 (p = 0.039) and IL-1 β (p = 0.016). LPS treatment significantly upregulated the expression of the anti-inflammatory cytokine IL-10 (p = 0.0005), which was reduced by Rosi (LPS + Rosi group, p = 0.0007) (Fig. 1-E).

### The effects of Rosiglitazone on LPS-induced trophoblast apoptosis and proliferation

**Figure 2:**
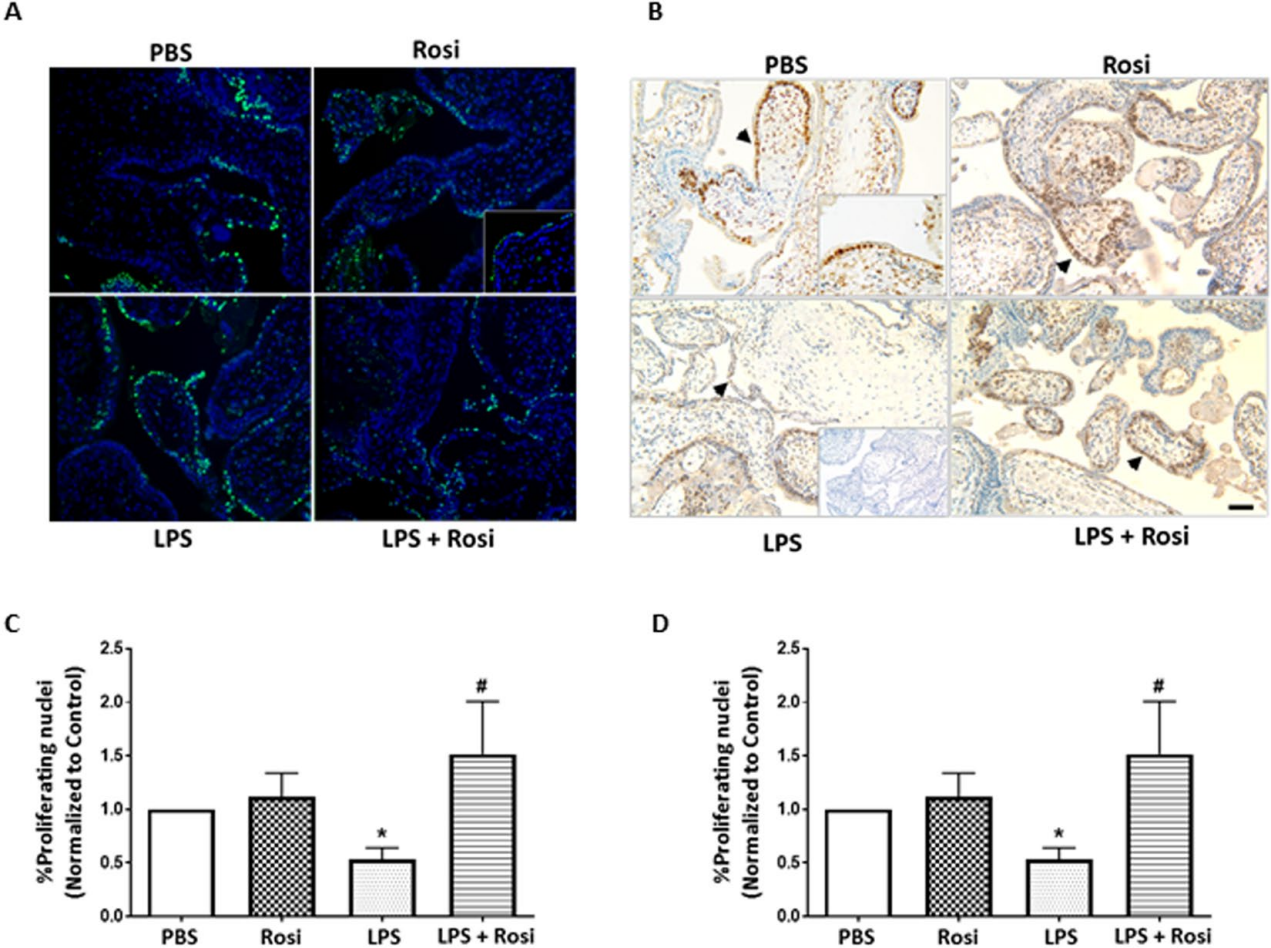
Rosiglitazone treatment reduced LPS induced apoptosis and increased proliferation 1^st^ trimester human placental trophoblast. Representative image showing (**A**) TUNEL staining and (**B**) for positive PCNA staining across the 4 treatment groups, positive nuclei identified by green nuclei and brown color due to oxidation of DAB (black arrowheads) respectively. (**C**) Percentage of apoptotic trophoblast cells identified by TUNEL staining and (**D**) Percentage of proliferating trophoblast nuclei after 24 hrs of treatment. Insets show the IgG control (**B**) and 40X magnification (**A**,**B**). n = 4–8, ‘*’ indicates significance at p < 0.05 when compared to the PBS control group, ^‘#’^ indicates significance at p < 0.05 when compared to the LPS treated group. Scale bar: 50 µm.

Pro-inflammatory cytokines that included TNF-α and IL-8 reduce proliferation and induce apoptosis in hematopoietic and mesangial cells^39,40^. To evaluate if LPS induced inflammatory cytokines had similar effects in trophoblast cells, the rate of apoptosis was assessed by TUNEL assay. The LPS treated explants (LPS group) showed a significantly higher percentage of apoptotic trophoblast nuclei (p = 0.02) at the end of culture when compared to the PBS-treated control explants. The percentage of apoptotic nuclei in explants treated with LPS + Rosi was significantly lower when compared to the LPS group (p = 0.03) and were comparable to the PBS control group (Fig. 2A,C). The percentage of apoptotic nuclei in the Rosi and PBS control groups was not significantly different.

The rate of proliferation in treated tissues was assessed by systematically counting the trophoblast nuclei stained positively for PCNA antigen over the total number of trophoblast nuclei. PCNA is an auxiliary protein involved in DNA replication and used as a marker for cell proliferation^41^. The explants treated with LPS had a significantly lower percentage of positively stained trophoblast nuclei (p = 0.01) when compared to the PBS group. Treatment with Rosi increased the rate of proliferation when compared to both the LPS and PBS treated explants. The values reached statistical significance for the comparison between LPS and LPS + Rosi groups (p = 0.02) (Fig. 2B,D). The proliferation rates in the Rosi and PBS groups were not significantly different.

### The effects of Rosiglitazone on LPS-mediated reduction of villous trophoblast differentiation

**Figure 3:**
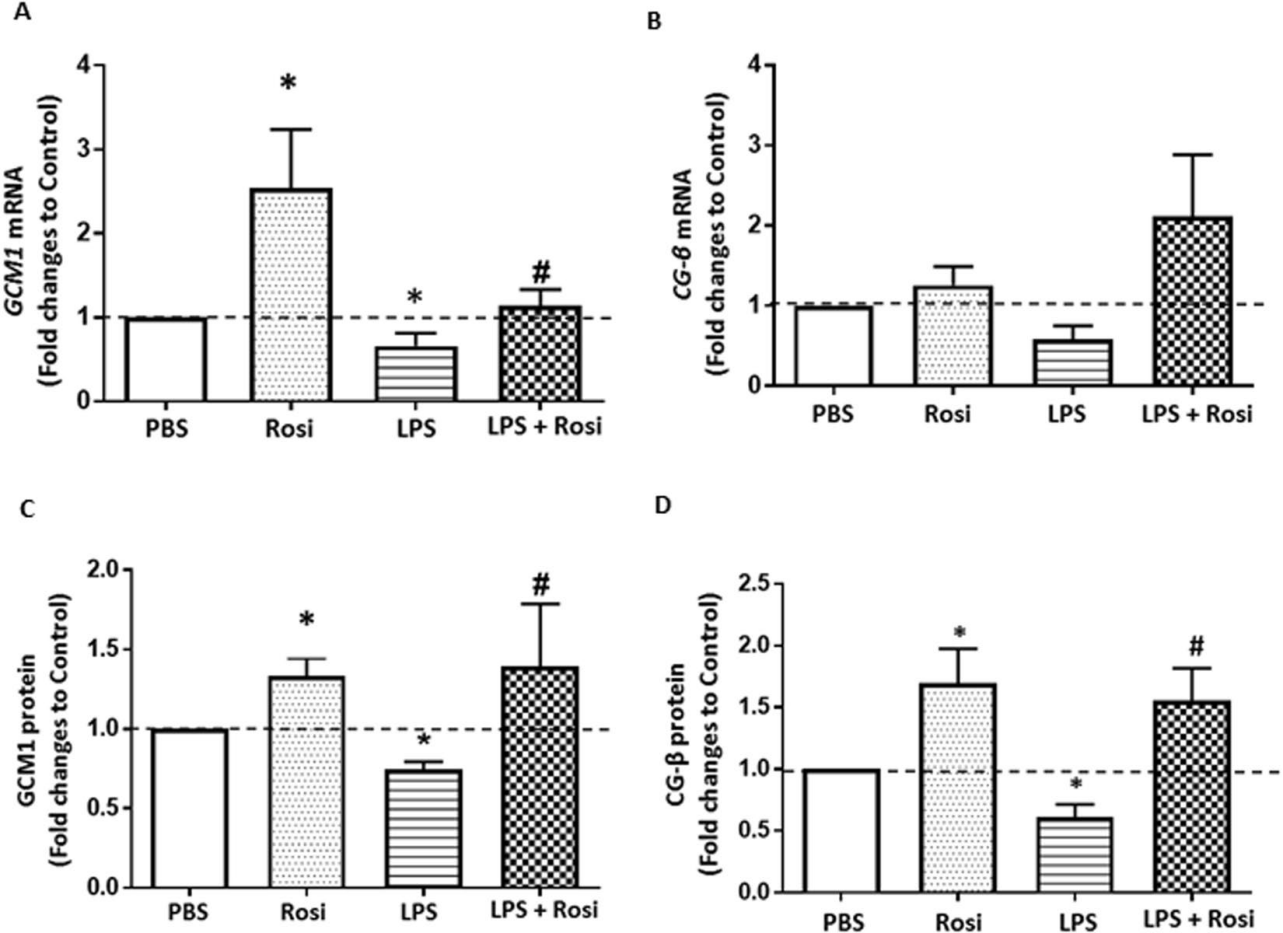
Rosiglitazone reversed LPS mediated reduction in trophoblast differentiation marker expression. Expression of trophoblast differentiation markers GCM1 and CG-β was assessed at mRNA level by qPCR (**A**,**B**) and protein level using western blotting and ELISA (**C**,**D**). LPS exposure downregulated expression of both mRNA and protein levels of GCM1 and CG-β which were induced by Rosiglitazone treatment. n = 4–8, ‘*’ indicates significance at p < 0.05 when compared to the PBS control group, ^‘#’^ indicates significance at p < 0.05 when compared to the LPS treated group.

To evaluate effects on villous trophoblast (VT) differentiation, expression of known differentiation markers was measured in 1^st^ trimester villous explants. Compared to the control group, exposure to LPS significantly reduced the expression of *Gcm1* mRNA (40% reduction, p = 0.04), measured by qPCR, and protein, (p = 0.01) semi-quantified by western blotting (Fig. 3A,C). Activation of PPARγ via Rosi reversed this reduction and significantly induced expression of GCM1 at both mRNA (p = 0.01) and protein levels (p = 0.05) (Fig. 3A,C) similar to previous reports in the BeWo cell line^42^. LPS did not significantly change β-hCG mRNA expression (p = 0.06 vs LPS group), but significantly lowered protein levels as measured by ELISA in the culture media (p = 0.009 vs LPS group) (Fig. 3B,D). PPARγ activation also did not significantly upregulate β-hCG mRNA, however, secretion of β-hCG (p = 0.002) was significantly upregulated (Fig. 3B,D). Treatment with Rosi alone also significantly induced protein expression of both GCM1 (p = 0.03) and β-hCG (p = 0.002) when compared to PBS group explants (Fig. 3A–D). To further delineate the effects of LPS on villous trophoblast differentiation, expression of protein specific glycoprotein 1 (PSG1) – and Syncytin 1 (SYN) was studied as markers of villous trophoblast fusion^43,44^. LPS exposure did not show any effects on PSG1 or SYN expression (Supplementary Fig. S1).

### The effects of Rosiglitazone on LPS-induced extravillous trophoblast invasion

Poor trophoblast invasion is associated with placental disorders such as PE and attributed to abnormal differentiation, leading to failure to remodel the uterine spiral arteries. To determine if altered expression of differentiation markers affected extravillous trophoblast (EVT), their invasion capacity was assessed using a Matrigel invasion assay^20^.

**Figure 4:**
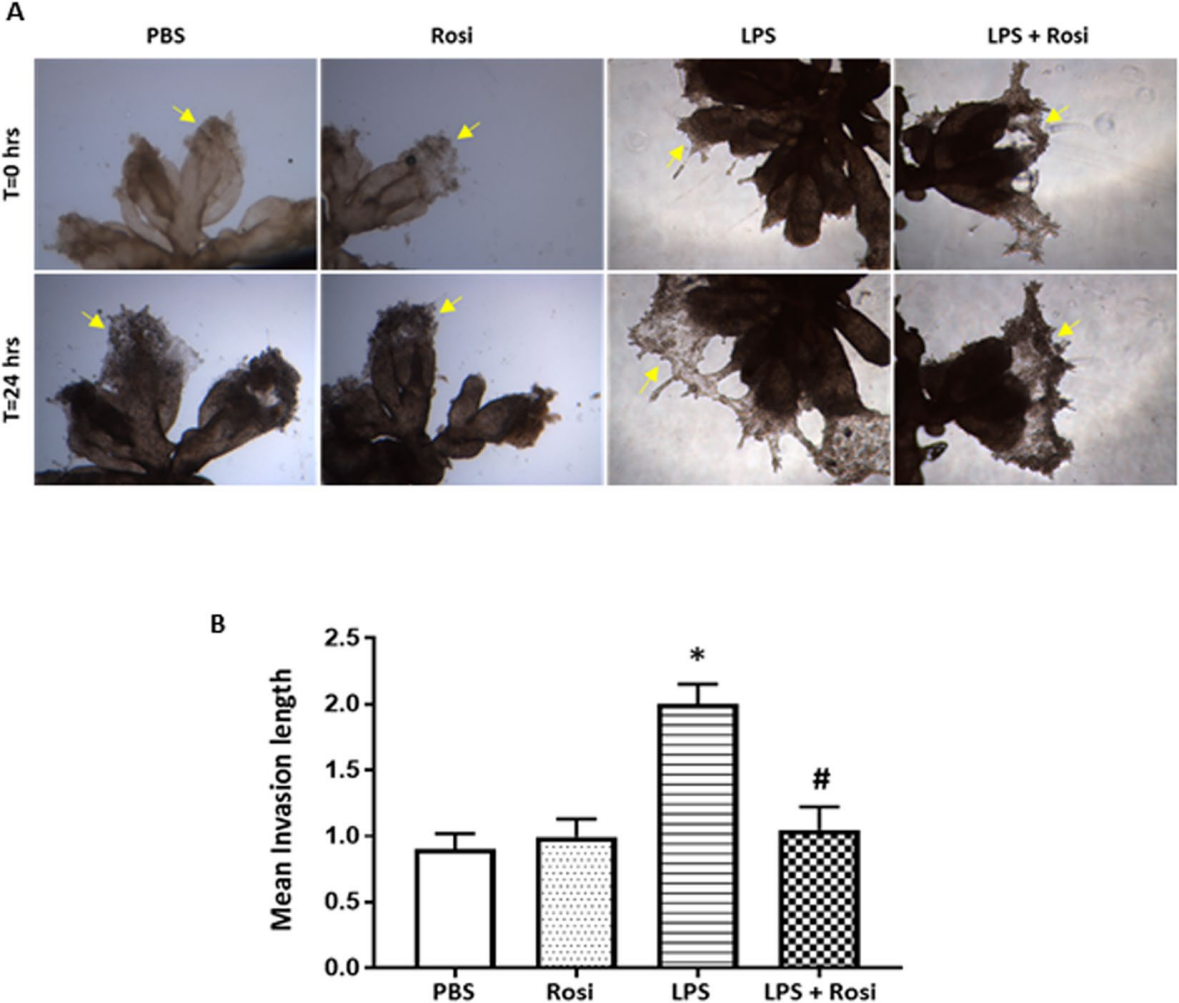
LPS increased invasion in 1^st^ trimester human placental explant cultures. Villous clusters with EVT tips were cultured on matrigel and treated for a period of 48 hrs. (**A**) Representative images for mean length of invasion over the culture period. (**B**) Graph shows mean length of invasion in different groups measured after 24 hrs of treatment. n = 5, ‘*’ indicates significance at p < 0.05 when compared to the PBS control group, ^‘#’^ indicates significance at p < 0.05 when compared to the LPS treated group. Magnification: ×100.

First trimester anchoring villi containing EVT cells (columns) were cultured on Matrigel at physiological 3% O_2_ for 24 hrs and the length of outgrowth was quantified, as described in the Methods section. LPS exposure induced invasion, with an average length of outgrowth into the Matrigel of 2.0 ± 0.4 µm (Mean ± SD), which was significantly longer compared to the PBS control group: 0.9 ± 0.2 µm (p = 0.0016) (Fig. 4A,B). In the presence of Rosi (LPS + Rosi group), the outgrowth length was maintained at a mean length of 1.0 ± 0.4 µm, comparable to that of the PBS group. The mean outgrowth length in the Rosi group was similar to the PBS group (p = 0.19).

### The effects of LPS on invasion in the extravillous-like trophoblast cell line HTR-8/ SVneo

Gestational age and oxygen concentration play critical roles in trophoblast invasion and could impact observations in the 1^st^ trimester placenta model^45^. To validate that effects on invasion were independent of O_2_ levels and gestational age, the trophoblast cell line HTR-8/SVneo was used at ambient oxygen levels to verify the initial results.

**Figure 5:**
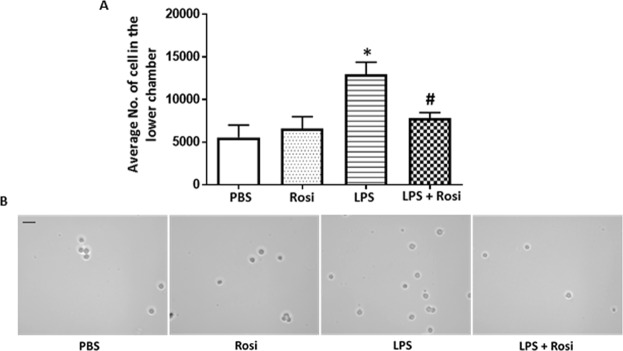
Rosiglitazone reduced LPS mediated invasion in trophoblast cell line HTR- 8/SVneo. (**A**) Graph shows the average number of cells counted in the lower chamber in the matrigel invasion assay with HTR-8/SVneo cells. Treatment with LPS significantly increased number of cells in the lower chamber which reduced when treated with Rosiglitazone. (**B**) Representative images show cells in the lower chamber in wells with respectively labelled treatments. n = 6, ‘*’ indicates significance at p < 0.05 when compared to the PBS control group, ^‘#’^ indicates significance at p < 0.05 when compared to the LPS treated group. Scale bar: 50 µm.

**Figure 6:**
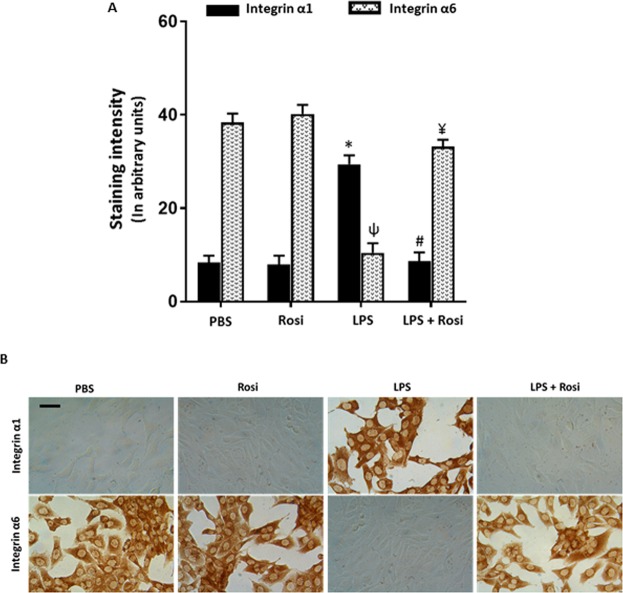
Rosiglitazone prevented LPS mediated integrin switching in trophoblast cell line HTR- 8/SVneo. HTR-8/SVneo cells were stained for assessing the expression of integrins α1 and α6. (**A**) Graph shows mean staining intensity for α1 and α6 across the different treatment groups. (**B**) Representative images showing α1 and α6 integrin staining across different treatments, showing the integrin switch in the LPS group which was prevented in the LPS + Rosi group. n = 6, ‘*’, ^‘ψ’^ indicates significance at p < 0.05 when compared to the PBS control group for α1 and α6 respectively, ^‘#’, ‘¥’^ indicates significance at p < 0.05 when compared to the LPS treated group for α1 and α6 respectively. Scale bar: 50 µm.

Exposure to LPS significantly increased the number of HTR-8/SVneo cells invading Matrigel (12,974 ± 2238 (Mean ± SE), p = 0.01), compared to the PBS control group (5560 ± 1440) (Fig. 5). Presence of Rosi in the culture significantly reduced these effects (LPS + Rosi group: 7819 ± 488, p = 0.05), compared to the LPS group (Fig. 5). The number of invading cells was comparable in the PBS, Rosi only and LPS + Rosi groups. Trophoblast cells undergoing differentiation to the invasive phenotype switch integrin expression from integrin α6 to α1^46^. The LPS treated HTR-8/SVneo cells were stained with antibodies against integrins α1 and α6 and the staining intensity semi-quantified using image analysis (Fig. 6). HTR-8/SVneo cells treated with LPS had a significantly higher expression of integrin α1 (LPS vs PBS: 29.4 ± 1.6 vs 8.3 ± 1.4 arbitrary units (AU), (Mean ± SD), p < 0.0001) and reduced expression of integrin α6 (LPS vs PBS: 10.4 ± 2.0 vs 38.3 ± 1.9 AU, p < 0.0001) when compared to the PBS controls (Fig. 6A). Treatment with Rosi prevented integrin switching and the LPS + Rosi group had significantly lower expression of integrin α1 (LPS + Rosi vs LPS: 8.6 ± 1.9 vs 29.4 ± 1.6 AU, p < 0.0001) and higher expression of α6 (LPS + Rosi vs LPS: 33.1 ± 1.5 vs 10.4 ± 2.0 AU, p < 0.0001) when compared to the LPS treated group. The expression of both integrin α1 and α6 in the LPS + Rosi, Rosi and PBS groups was comparable (Fig. 6).

### Requirement of NF-κB signaling for LPS-mediated induction of trophoblast invasion

**Figure 7:**
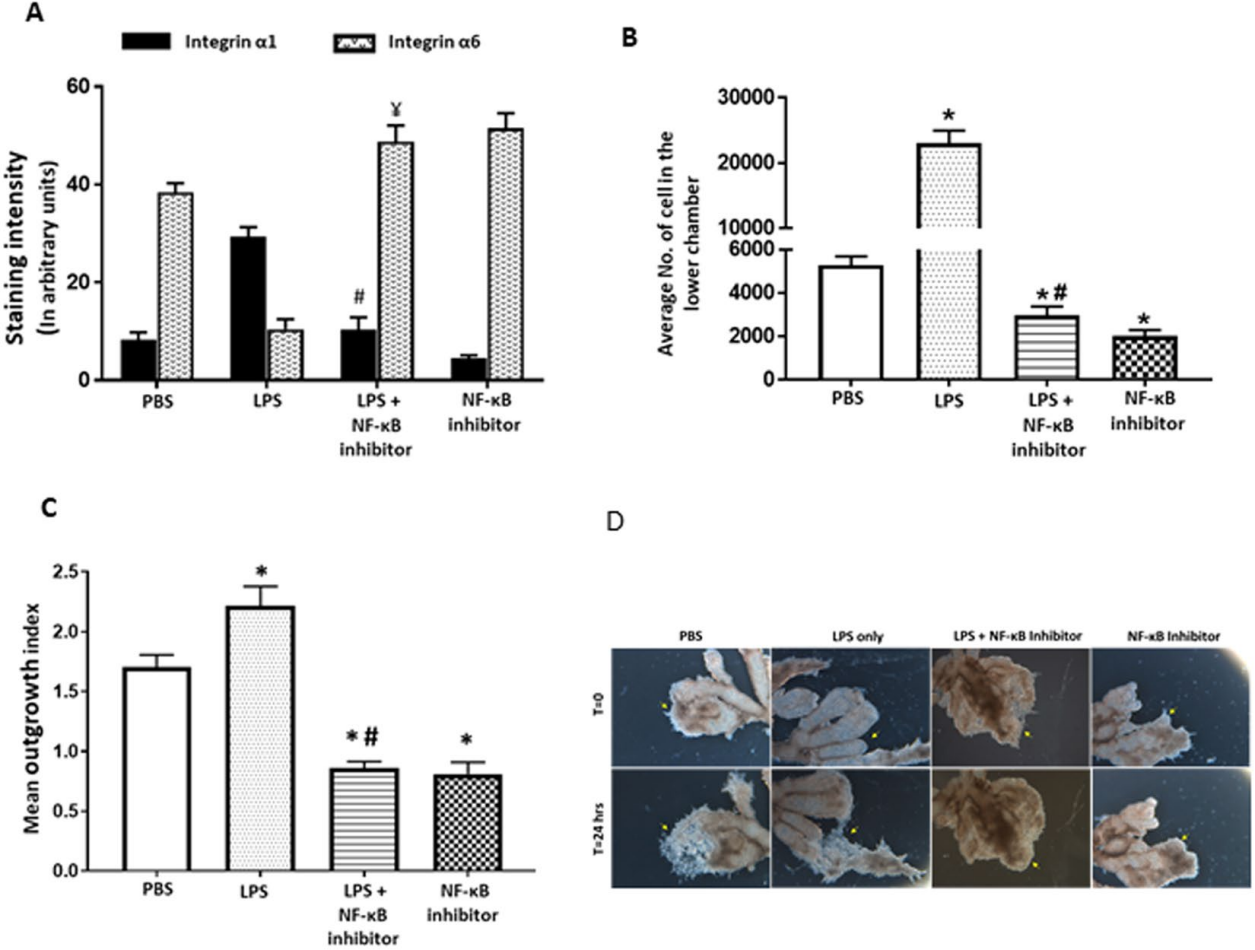
LPS mediated increase in invasion was prevented by inhibition of NF-κB activity. (**A**) HTR-8/SVneo cells were stained for assessing the expression of integrins α1 and α6. Graph shows mean staining intensity for α1 and α6 across groups. (**B**) Graph shows the average number of cells counted in the lower chamber in the matrigel invasion assay with HTR-8/SVneo cells treated with LPS + NF-κB inhibitor. (**C**) Graph shows mean length of invasion in different groups measured after 24 hrs of treatment showing reduction in LPS induced invasion in presence of NF-κB inhibitor. (**D**) Representative image showing effects of NF-κB inhibitor on invasion in explant villous clusters with EVT tips. n = 4, ^‘*’, ‘ψ’^ indicates significance at p < 0.05 when compared to the PBS control group for α1 and α6 respectively, ^‘#’, ‘¥’^ indicates significance at p < 0.05 when compared to the LPS treated group for α1 and α6 respectively.

Both LPS and Rosiglitazone modulate inflammation through the transcription factor NF-κB^47^. To evaluate whether the effects of LPS on trophoblast invasion were mediated by NF-κB, HTR-8/SVneo cells were treated with LPS ± the specific NF-κB inhibitor (TPCA-1). Integrin sub-type switching and Matrigel invasion were quantified. NF-κB inhibitor prevented differentiation and invasion-associated integrin switching in response to LPS. The staining intensity of α1 in the LPS + NF-κB inhibitor group was significantly lower (LPS + NF-κB inhibitor vs LPS: 10.3 ± 2.4 vs 29.4 ± 1.6 AU, p < 0.0001), while α6 was significantly higher compared to the LPS group (LPS + NF-κB inhibitor vs LPS: 48.7 ± 3.4 vs 10.4 ± 2.0 AU, p < 0.0001) (Fig. 7A). Matrigel invasion was reduced in the LPS + NF-κB inhibitor group compared to the LPS group (p = 0.002) and the PBS group (p = 0.004). Cells treated with only TPCA-1 had significantly lower numbers of invading cells than either the LPS group (p = 0.0022) or the PBS control group (p = 0.0043) (Fig. 7B). Comparable results were obtained using the first trimester explant invasion assay. Explants treated with LPS + NF-κB inhibitor showed significantly less outgrowth (Fig. 7C,D) than the LPS or PBS control group (p < 0.0001 vs LPS and vs PBS group). Treatment with TPCA-1 alone significantly inhibited explant outgrowth (p < 0.0001 vs LPS and vs PBS group), suggesting a basal requirement for NF-κB in trophoblast invasion.

### The effects of LPS and Rosiglitazone on interactions between NF-κB and active RNA Pol II

**Figure 8:**
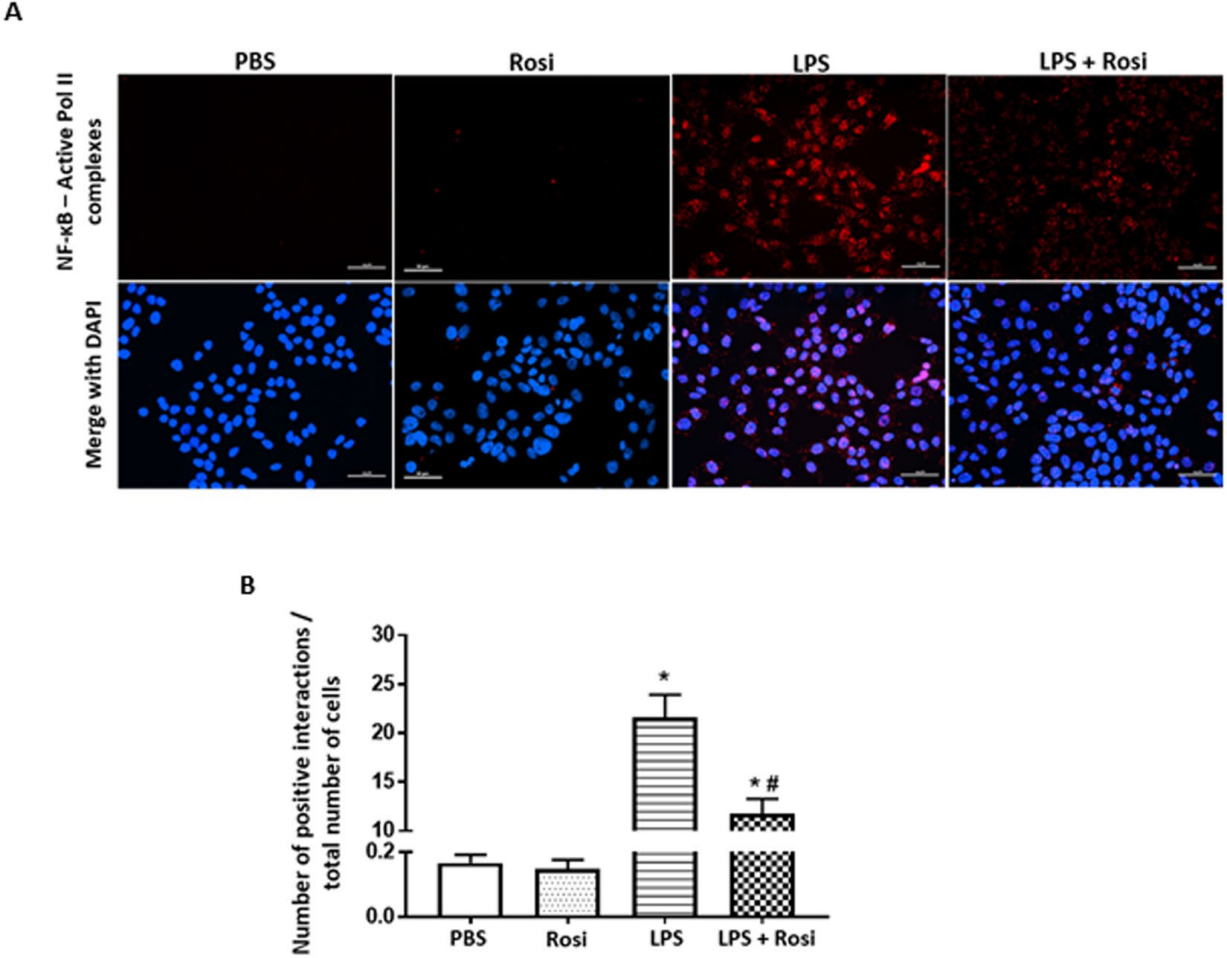
Rosiglitazone reduces LPS induced NF-κB activity in HTR-8/SVneo cells. HTR8/SVneo cells were stained for co-localizing NF-kB and active Pol II using the proximity ligation assay. (**A**) Representative images showing colocalization events across the four treatment groups. (**B**) Graphs shows quantitative analysis for number of co-localization events with increase in the number of events in the LPS treatment group. n = 6, ^‘*’,‘ #’^ indicates significance at p < 0.05 when compared to the PBS control group and the LPS treated group respectively. Scale bar: 50 µm.

The transcriptional activity of NF-κB in response to LPS was evaluated by using the proximity ligation assay, which enables the detection and visual semi-quantification of protein – protein interaction events *in situ*^48^. A positive reaction is seen only when two targeted proteins are in close proximity. The interaction between NF-κB and active RNA polymerase II protein (Pol II) was measured and used as an indicator of NF-κB transcriptional activity. LPS exposure increased interaction between NF-κB and active RNA Pol II (increased number of red spots in Fig. 8A- third panel from left, 8B, p < 0.0001 vs PBS group). This interaction was reduced in cells treated with LPS + Rosi (reduced number of red spots in Fig. 8A- right panel, 8B, p = 0.0007 vs LPS group). The number of proximity events in PBS and Rosi groups was comparable.

## Discussion

Several studies have explored the effects of inflammation on the placenta in rodent and human trophoblast cell models^49,50^. Studies in human models have mostly focused on cytokine secretion and related inflammatory pathways with little information about effects on trophoblast physiology^15,50^. Furthermore, most studies relied on the use of isolated primary trophoblast cells, which rapidly undergo terminal differentiation in culture, or trophoblast cell lines that show different responses to the same inflammatory stimulus^51–53^. These characteristics limit the usefulness of those models. Here, a 1^st^ trimester placental explant model was employed that currently is most representative of the *in vivo* condition as it specifically maintains tissue integrity, which is important for trophoblast turnover^20^. Using LPS exposure as a model of inflammation in 1^st^ trimester placental explants, we highlight a previously undescribed role for NF-κB in trophoblast invasion, and a gate keeper function for PPARγ in the regulation of human placental tissue inflammation, trophoblast maintenance and differentiation.

The secretion of inflammatory cytokines from 1^st^ trimester villous placental explants exposed to LPS and Rosiglitazone was assessed to confirm the validity of the model. Increased expression of inflammatory cytokines (as well as IL-10) confirmed the induction of inflammation. Further, reduced secretion of inflammatory cytokines and IL-10 due to Rosiglitazone confirmed the anti-inflammatory activity of PPARγ^15,50^. Upregulation of the anti-inflammatory cytokine IL-10 due to LPS exposure of trophoblast was previously reported and is considered a defense mechanism against the ensuing inflammation^52–59^. Our results confirm a similar mechanism in the 1^st^ trimester human placenta. LPS-induced inflammation altered cell proliferation and apoptosis in immune and endothelial cells, but the available literature on cell cycle in trophoblast is controversial^54,55^. LPS exposure did not alter proliferation in isolated primary trophoblast cells, but increased apoptosis after three days of culture^15,56^. JEG-3 choriocarcinoma cells showed increased apoptosis and reduced proliferation rates, while HTR-8 SV/neo cells showed increased proliferation and no effect on apoptosis after exposure to either LPS or inflammatory proteins^57–59^. These observations can be attributed to the features of immortalized cell lines, or their origin (HTR-8 SV/neo are extravillous, whereas JEG-3 cells are villous in origin) and the tendency of primary cell isolates to exit the cell cycle and terminally differentiate in culture due to the loss of tissue integrity. In the villous explant model, inflammation significantly increased apoptosis and reduced proliferation, suggesting an overall negative effect on trophoblast cell cycle. Activation of PPARγ restored both apoptosis and proliferation rates to control levels, indicating a protective effect. PPAR-γ activation has both pro and anti-apoptotic and proliferative effects, depending on the activating ligand (including Rosiglitazone), type of model system and presence of adaptor proteins^60,61^. However, since Rosiglitazone alone did not alter apoptotic or proliferation rates, it is likely that PPARγ does not directly regulate those processes. Instead, it acts indirectly through downregulation of the inflammation cascade to restore or maintain trophoblast physiological integrity.

To determine if the altered cell cycle caused any changes in trophoblast function, expression of differentiation markers GCM1 and β-hCG was assessed. Both GCM1 and β-hCG are important for EVT and VT lineage differentiation. GCM1 knockdown in 1^st^ trimester human placental explants significantly inhibits invasion in EVT’s and syncytialization in VT^20^. Similarly, β-hCG regulates EVT invasion and its knockdown reduces fusion in BeWo cells^62–64^. Here for the first time, inflammation reduced both markers in VT explants, indicating dysregulation of trophoblast differentiation, which was rescued by PPARγ activation. Since treatment with Rosiglitazone induced expression of both GCM1 and β-hCG (Rosi group), as previously reported in the BeWo choriocarcinoma cell line^21,42^, the results also confirmed a role for PPARγ in trophoblast differentiation, possibly through independent pathways.

Despite changes in GCM1, expression of PSG1 and SYN, proteins associated with syncytialization in VT’s, was unaffected by both inflammation and Rosiglitazone, indicating no effects downstream of GCM1 on VT differentiation. Baczyk *et al*. noted effects of GCM1 knockdown on syncytialization in 1^st^ trimester VT explants after 3 days of culture^20^. Based on their observation, we suspect that the 24-hour exposure period in our study would be insufficient to detect changes in VT differentiation. Additional studies are required to further decipher both the VT differentiation machinery and the effects of inflammation. Interestingly, elevated inflammation increased trophoblast outgrowth from explants, suggesting an increase in EVT differentiation after 24 hours of treatment. These results were counterintuitive to the observed decrease in GCM1 and β-hCG, and contradictory to earlier reports in isolated primary trophoblasts. Primary cells do not proliferate rather always terminally differentiate in culture which can explain the differences in results obtained^15,50^. Due to the technical limitations of the invasion assay, we were unable to assess GCM1 and β-hCG specifically in the invaded cells, which is a limitation of the current study and requires future validation. Nevertheless differential regulation of pathways involved VT and EVT differentiation has been previously suggested and support the results in this study^65^. Additionally, the differences in gestational age of tissues and culture conditions used for EVT and VT differentiation studies might have affected the data. The explants used for invasion were younger in gestational age 5–7 weeks v-s the10–12 week old tissues used for villous culture as at an older gestation EVT cell columns are rare and invasion capacity is potentially reduced^66^. The invasion assays were performed at 3% O_2_ tension to mimic the physiological conditions during early gestation. Both gestational age and O_2_ tension have been previously shown to affect invasion and could influence our results^45,67,68^. To account for these effects, experiments were repeated with the HTR-8/SVneo cells. As in explants, inflammation increased HTR-8/SVneo cell invasion, which was reversed by Rosiglitazone. This was further supported by inflammation-induced integrin switching from α6 to α1 subunit expression, as found in the invasive phenotype of EVT cells^46^. Thus, we were able to show across two culture models that inflammation increases EVT invasion.

The increase in invasion due to inflammation was unexpected, as placental insufficiencies (often displaying high inflammation) are thought to stem from shallow EVT invasion^6,10^. Recent studies of trophoblast behavior suggest that under stress trophoblast cells regulate the balance between proliferation and terminal differentiation based upon the nature of stress encountered^69^. Under hypoxic stress, trophoblast cells were shown to reduce proliferation and increase differentiation^70^. The data presented here suggests similar behavior under inflammatory stress. We suggest that increased EVT invasion in early gestation could lead to depletion of the proliferative stem cell pool or disturb the balance between syncytiotrophoblast and EVT differentiation potentially contributing to placental insufficiency. Our data warrants further detailed studies of EVT and VT differentiation pathways independently under inflammatory stress to better understand the pathophysiology of placental insufficiency disorders. Further, PPARγ activation alone did not alter trophoblast invasion in explants or HTR-8 SV/neo cells (in contrast to previous studies conducted in isolated primary trophoblast cells), but restored inflammation induced invasion^71^. This suggests a complex role for PPARγ in trophoblast invasion, and an indirect regulatory role via inflammatory pathways (comparable to the apoptotic pathway) in the current model.

A common downstream target of LPS and Rosiglitazone is the transcription factor NF-κB, also reported to be involved in cell differentiation and cancer cell migration^72–74^. However, these roles have not been defined in the human placenta. To mechanistically link inflammation and trophoblast differentiation, NF-κB, activity was blocked selectively by inhibiting the activity of I-κB kinase which is required by NF-κB for its activity^75^. Exposure to the inhibitor also repressed LPS-induced invasion in EVT- explants and prevented the reduction in β-hCG secretion from villous explants, confirming that the effects of inflammation were in fact mediated by NF-κB. This aligned with the observations that PPARγ activation downregulated effects of inflammation, including NF-κB targets, TNFα, CCL5 and IL-1 β^76–79^. Indeed, Rosiglitazone via PPARγ has been shown to downregulate NF-kB activity^80,81^. To confirm this observation in our model, changes in NF-κB transcriptional activity were assessed using proximity ligation approach and involvement of NF-κB was confirmed. Interestingly, exposure to NF-κB inhibitor alone was sufficient to reduce invasion in both HTR-8/SVneo cells and tissues. The current data show for the first time a role of NF-κB in trophoblast differentiation. While we cannot rule out the involvement of PPARγ independent pathways in our model, our data provides proof for a potential molecular mechanism involving PPARγ – NF-κB axis linking inflammation and trophoblast differentiation.

The pregnancy pathologies PE, IUGR and PTB are associated with elevated inflammation, placental inflammatory cytokine secretion, trophoblast apoptosis and abnormal trophoblast differentiation. These activities are linked to decreased placental expression of GCM1 and β-hCG, as well as disrupted trophoblast invasion^9,10,82–84^. The present study provides evidence for cross-talk between inflammation and differentiation processes in the human trophoblast. Further investigation into the PPARγ – NF-κB axis will provide a better understanding of the relationship between inflammation and trophoblast differentiation/invasion and how it relates to placental pathologies.

## Materials and Methods

### Tissue collection

Human first trimester placental tissues (5–12 weeks) were obtained with written informed consent from healthy pregnant women following elective termination of pregnancy at the Michigan Family planning facility, Michigan, US and Morgentaler Clinic, Toronto, Canada. The Institutional Review Board (IRB) of Wayne State University and Mount Sinai Hospital (MSH) Research Ethics Board approved all consent forms and protocols used in this study and all experiments were performed in accordance with relevant guidelines and regulations.

### First trimester explant culture

The chorionic villi from 1^st^ trimester human placenta were micro-dissected under a microscope into small pieces (20–30 mg wet weight) as previously described in^85^ and cultured in 1 ml of Dulbecco’s Modified Eagle Medium – F12 (DMEM/F12) media, without phenol red (Life Technologies, USA) containing 10% Fetal bovine serum (FBS, Atlanta Biologicals, USA) and 1% Antibiotic-Antimycotic (Life Technologies, USA). The explants were treated in triplicates, by adding the respective treatments to the culture medium and then incubating at 8% O_2_, 37 °C for 24 hrs. After 24 hrs, the explants were weighed and snap frozen to be used later for either RNA extraction or protein analysis. One replicate was fixed in 4% paraformaldehyde (PFA, Fischer Scientific, USA) for 60 minutes and paraffin embedded to be used for immunohistochemistry. The media was frozen and used for ELISA.

### Cell culture

The HTR-8/SV NEO-8/SVneo cytotrophoblast cell line (passage number 25–35) was cultured on plastic in T-75 tissue culture flasks (Corning) in DMEM/F12 media (Life Technologies, USA) containing 10% Fetal bovine serum (Atlanta Biologicals, GA) and 1% Antibiotic-Antimycotic (Life Technologies, USA) in a humidified incubator at 5% CO_2_. Culture medium was replaced with serum-free medium prior to all treatments^86^.

### Explants and cell culture treatments

LPS (*Escherichia coli* 055: B5; Sigma-Aldrich, USA) was reconstituted in sterile 1X Phosphate buffered saline (PBS) (Life technologies, USA) and stored at −20 °C. Cell or villous explant cultures were treated by supplementing culture medium with 1 µg/ml LPS in presence or absence of 10 µM Rosiglitazone (Sellekchem, USA). Cells/explants treated with 1X PBS and 10 µM Rosiglitazone were used as controls. The treatment groups will be identified as outlined in Table 1. In addition to these, HTR-8/SVneo cells were also treated with 100 nM 2-[(Aminocarbonyl)amino]-5-(4-fluorophenyl)-3-thiophenecarboxamide (TPCA-1) (Tocris, UK), a specific inhibitor for NF-κB activity. HTR-8/SV neo were used from the same passage range of maximum of 10 passages. Cells were confirmed to express extravillous trophoblast markers by qPCR and immunohistochemistry to ensure cell identity (data not shown).

**Table 1:** Trophoblast explants/cell treatment groups and names.

Treatment	Group Name
LPS - 1 µg/ml	LPS/LPS
LPS - 1 µg/ml + Rosiglitazone- 10 µM	LPS + Rosi
1X PBS	Control
Rosiglitazone- 10 µM	Rosi

### RNA extraction and real time PCR

The explants/cells were lysed in 0.9 ml Qiazol (Qiagen, Germany). Total RNA was extracted (RNAeasy Plus Universal Mini kit, Qiagen, Germany) and all samples in equal amounts were simultaneously reverse transcribed using the RT synthesis kit from Bio-Rad per the manufacturer’s protocol (iScript Reverse Transcription Supermix, Bio-Rad Laboratories, USA). Real-time PCR was performed on the Bio-Rad CFX384 real time system in triplicates in 10 uL total reaction volume containing 10 ng of template cDNA, 5 µL of SYBR-green master mix (LuminoCT, Sigma-Aldrich, USA) and 500 nM of primers. The primers used for assessing the expression levels of target and housekeeping genes are outlined in Table 2. Data was analyzed using the delta delta CT method as described in^87^.

**Table 2:** List of primer sequences used for studying expression levels of trophoblast differentiation markers.

Gene Name	Gene Symbol	Sequence
Cytochrome - C 1*	*Cyc1*	5′-CAT CAT CAA CAT CTT GAG CC-3′
		5′-CAG ATA GCC AAG GAT GTG TG-3′
Tyrosine 3-Monooxygenase/Tryptophan 5-Monooxygenase Activation Protein Zeta*	*Ywhaz*	5′- CCG CCA GGA CAA ACC AGT AT -3′
		5′- ACT TTT GGT ACA TTG TGG CTT CAA -3′
TATA Box Binding Protein*	*Tbp*	5′-CAC ATC ACA GCT CCC CAC CA-3′
		5′-TGC ACA GGA GCC AAG AGT GAA-3′
Glial cell missing 1	*Gcm1*	5′-TGA ACA CAG CAC CTT CCT C-3′
		5′-CCA CTG TAA CTA CCA GGC AAT-3′
Human chorionic gonadotropin - beta	*Β-hCG*	5′-GGT TGA GGC TTC AGT CCA G-3
		5′-AGG GAG TAG GGT GTA GGA AG-3′

(* indicates genes used as housekeeping genes).

### Protein extraction and western blotting

Total proteins were extracted from the explants by homogenizing the explants in lysis buffer containing: 1% (w/v) SDS, 50 mM Tris, pH 6.8 and 10 mM NEM followed by heating the lysate at 100 °C for 10 mins. The lysates were then spun at 10,000 rpm for 15 mins and the supernatant was collected. Protein concentration was then determined using Pierce^©^ 660 nm protein assay reagent (Thermo Scientific, USA), per manufacturer’s instructions. Purified protein (30 µg) was separated on 12% SDS-polyacrylamide gel (TGX Stain Free Fastcast Acrylamide kit, Bio-Rad Laboratories, CA) and transferred on PVDF membrane (Bio-Rad Laboratories, USA). Membranes were blocked with 5% skimmed milk in Tris-buffer saline (TBST) containing 0.1% (v/v) Tween for 1 hr at room temperature (RT) and incubated over night at 4 °C with the anti-GCM1 antibody (1:3000) (Aviva System Biology, UK). The membranes were washed with TBST and incubated with a HRP-conjugated secondary antibody (Cell signaling) for 1 hr. at RT. The antibody binding was detected using the Western Lightning® ECL Pro detection kit (Perkin Elmer, USA). Signals were visualized using a ChemiDoc Imaging System (Bio-Rad Laboratories, USA) and Image Lab V.5.1 software (Bio-Rad Laboratories, USA). Densities of immunoreactive bands were measured as arbitrary units by ImageJ software. Protein levels were normalized to a housekeeping protein β-actin (1: 20,000; Abcam, UK).

### Enzyme Linked Immunosorbent Assay

The media collected from placental explant cultures was assayed for levels of secreted inflammatory cytokines – IL-6, IL-8, IL-1 β, TNF-α, CCL5 using the Duo Set ELISA development kits (R&D Systems, USA) as per the manufacturers protocol. The levels of CG-β were assayed using the Beta-Human Chorionic Gonadotrophin (β-hCG), free (Human) - ELISA Kit (Phoenix Pharmaceuticals, USA) again as per the manufacturer’s protocol. The optical density of the final colored reaction product was measured at 450 nm using multispectral UV/VIS plate reader (Bio-Tek, VT). Standard curves were used to calculate protein in content in the samples. The level of proteins detected was normalized over the wet weight of the explant to obtain the amount of protein secreted per milligram of explant tissue. The data was then normalized to control treatment to take sample to sample variations into account. The data was analyzed for statistical difference using the Graph pad prism 7.0 software.

### Immunohistochemistry

Immunostainings of placental villi were performed as described before^88^. Briefly, the sections were deparaffinized and rehydrated, followed by antigen retrieval using Dako Target retrieval solution (Agilent-DAKO, USA). The intrinsic peroxidase activity was then quenched by incubating the sections with 3% Hydrogen peroxide (Fisher Scientific, MA) for 30 mins at RT, followed by a wash with 1X PBS. The sections were then incubated overnight at 4 °C with anti-PCNA (Santa Cruz, TX) or 10 μg/ml nonimmune Rabbit IgG (Jackson Immunoresearch, PA) (used as a negative control). The following day, the slides were washed 3 times (5 minutes/wash) with 1X PBS containing 0.1% Tween 20. The samples were then incubated for 30 min with a peroxidase-conjugated polymer coupled to anti-rabbit IgG (EnVision Systems Peroxidase, Agilent-DAKO, USA). The peroxidase was visualized with 3,3-diaminobenzidine (DAB, Agilent-DAKO, USA) and hydrogen peroxide for 5 min. Tissues were counterstained with hematoxylin, dehydrated and were cover slipped. The staining was visualized using Nikon Eclipse 90i epifluorescence microscope (Nikon Inc., Japan) and the images were analyzed using ImageJ software.

HTR-8/SVneo cells were fixed with 4% PFA in 1X PBS for 10 mins at RT, washed 3 times with 1X PBS (5 mins/wash) and permeabilized with PBS containing 0.1% Triton-X100 for 10 mins. Cells were then incubated overnight at 4 °C with various primary antibodies against: anti-Integrin alpha 1 (EMD Millipore, USA), and anti-Integrin alpha 6 (Cell Signaling Technologies, USA) or 10 μg/ml of nonimmune mouse serum (Jackson Immunoresearch, PA), the next day cells were washed 3 times with 1X PBS (5 mins/wash) and incubated with peroxidase-conjugated polymer coupled to anti-mouse IgG (EnVision Systems Peroxidase, Agilent - DAKO, USA). The peroxidase was visualized with 3,3-diaminobenzidine (DAB, Agilent - DAKO, USA) and hydrogen peroxide for 5 min. The cells were then imaged using the Leica DM IRB epifluorescence microscope (Leica, Germany), and images were captured using a Hamamatsu Orca digital camera (Hamamatsu Corp, Japan). All samples were stained similarly to avoid staining bias.

To quantify the staining intensity, monochromatic bright field images of the antibody/DAB stained cells were obtained at ×400. Before imaging the brightness was adjusted in a region of each slide devoid of tissue/cells by setting the gray level to 255. Using Simple PCI imaging software (Hamamatsu Corp. Japan), 5 random fields were imaged per well and the mean gray level was determined. The intensity from 5 images fields was averaged to obtain the mean intensity per well and the intensity from 3 wells was combined to get the average intensity per treatment.

### Matrigel invasion assay

For placental explants, individual clusters of 6–8week old villi were dissected under a stereomicroscope and verified for the presence of extravillous trophoblasts (EVT’s) on the villous tips. These clusters were cultured on Millicell-CM inserts (12-mm diameter, 0.4-μm pores; EMD Millipore, USA) precoated with 0.2 mL of undiluted Matrigel (Corning, USA) in a 24-well culture plate for a total of 72 hours. The outer chamber contained 500 μL DMEM/F12 (Life Technologies, USA) without serum, medium supplemented with 10% Fetal bovine serum (Atlanta Biologicals, USA) and 1% Antibiotic-Antimycotic (Life Technologies, USA). The inner chamber contained approximately 200 μL of the same medium. The explants were treated by supplementing the media with respective drugs. The explants were imaged every 24 hrs for 48 hrs using Hamamatsu Orca digital camera (Hamamatsu Corp, Japan) and the outgrowths were measured using the ImageJ software. The mean outgrowth index was calculated by dividing the mean invasion length per explant after 24 hrs of treatment over its mean invasion length before starting the treatment. Each treatment was performed in duplicates for every tissue, and the experiment was repeated 3 times.

For assessing invasion of cell line, HTR-8/SV Neo cells (100,000) were cultured with treatments outlined in Table 1 on matrigel in 6.5 mm Transwell inserts as previously reported in^86^. Briefly, 15 µL of undiluted growth factor reduced matrigel was added onto the membrane and the inserts were placed into 24-well culture plates and incubated at 37 °C for 1 hr. for polymerization. After gel formation, the lower chamber was filled with 500 ml of serum-free DMEM/F12 medium and approximately 100,000 cells were cultured at 20% O_2_ & 37 °C for 24 hrs on the matrigel in 100 µL of medium. Cells that penetrated the matrigel and populated the lower chamber were detached using 500 µL Trypsin-EDTA solution (Life technologies, USA). The number of invading cells in response to treatment was quantified by microscopic imaging of 5 random fields per well and averaging the number of cells counted per field. Each treatment was performed in duplicates and the entire experiment was repeated 3 times. The calculation was performed by combining the average number of cells for each treatment across all experiments.

### TUNEL assay

For histological evaluation and quantification of apoptosis in paraffin embedded sections DNA strand breaks were detected by terminal deoxynucleotidyl transferase (TdT)-mediated dUTP nick end-labeling (TUNEL), using a fluorescein-based *in situ* cell death detection kit (Roche Applied Science, USA), per the manufacturer’s instructions. Sections were imaged with a Nikon Eclipse 90i epifluorescence microscope (Nikon Inc., Japan). The apoptotic trophoblast cells (TUNEL-positive nuclei) were counted at 200× from 5 random fields on each section from three samples for each treatment, along with the total number of nuclei (DAPI-labeled) to calculate the percentage of TUNEL/DAPI-labeled nuclei (TUNEL index). Sections subjected to treatment without TdT served as negative controls. The calculation was performed by averaging counts for four fields of each specimen from duplicate samples of at least three independent experiments.

### Statistical analysis

All data are shown as Mean ± S.E.M. The treatment groups were first normalized to the respective vehicle control and then analyzed. T-test, one-way ANOVA followed by Tuckey’s post-hoc test was performed to analyze differences between treatment using the GraphPad Prism 7.0 software. An effect was considered significant when p < 0.05.

## Supplementary information

Supplementary file
