# Supplementary material for: Rosiglitazone blocks first trimester in-vitro placental injury caused by NF-κB-mediated inflammation
Source: Sci Rep. 2019 Feb 14;9:2018. doi: 10.1038/s41598-018-38336-2 (PMC6376060; doi:10.1038/s41598-018-38336-2)
Supplement: Supplementary file 1 — Supplementary file [file 41598_2018_38336_MOESM1_ESM.docx]

**Title:** Rosiglitazone blocks first trimester *in-vitro* placental injury caused by NF-κB-mediated inflammation

**Authors:**

LK: Leena Kadam^1,2^,

BK: Brian Kilburn^1^,

DB: Dora Baczyk^3^,

HK: Hamid Reza Kohan-Ghadr^4^,

JK: John Kingdom^3^,

SD: Sascha Drewlo^4, *^

1: Department of Obstetrics & Gynecology, Wayne State University School of Medicine, Detroit, MI, USA.

2: Department of Physiology Wayne State University School of Medicine, Detroit, MI, USA.

3: The Research Centre for Women's and Infant's Health, Lunenfeld Tanenbaum Research Institute, Mount Sinai Hospital, University of Toronto, Toronto, Canada.

4: Department of Obstetrics, Gynecology and Reproductive Biology, College of Human Medicine, Michigan State University, Grand Rapids, MI, USA

**Supplemental figure 1.**

**
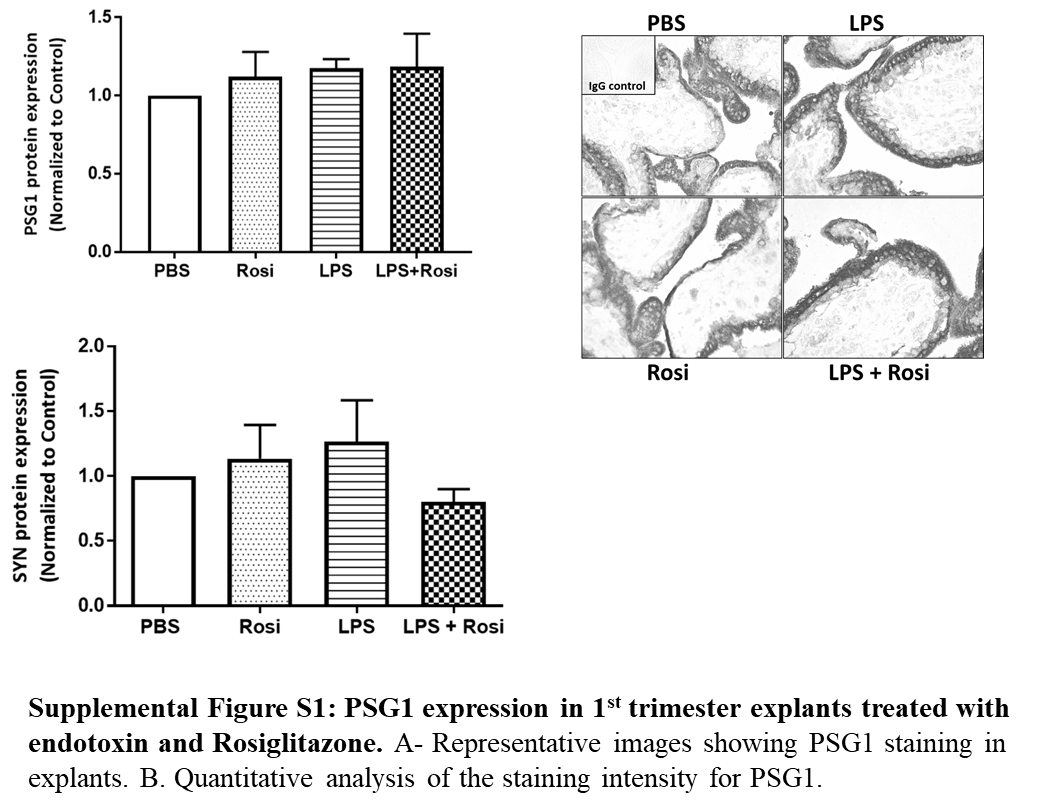
**

**
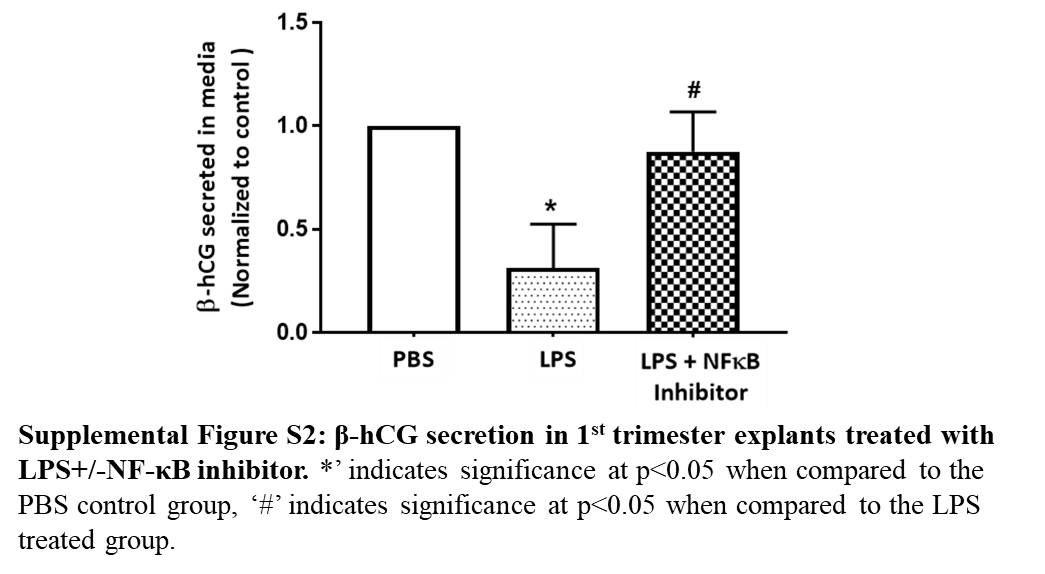
Supplemental Figure 2.**
